# Auditory roughness elicits defense reactions

**DOI:** 10.1038/s41598-020-79767-0

**Published:** 2021-01-13

**Authors:** Marine Taffou, Clara Suied, Isabelle Viaud-Delmon

**Affiliations:** 1 Institut de Recherche Biomédicale des Armées, 91220 Brétigny-sur-Orge, France; 2 CNRS, Ircam, Sorbonne Université, Ministère de la Culture, Sciences et Technologies de la Musique et du son, STMS, 75004 Paris, France

**Keywords:** Neuroscience, Cognitive neuroscience

## Abstract

Auditory roughness elicits aversion, and higher activation in cerebral areas involved in threat processing, but its link with defensive behavior is unknown. Defensive behaviors are triggered by intrusions into the space immediately surrounding the body, called peripersonal space (PPS). Integrating multisensory information in PPS is crucial to assure the protection of the body. Here, we assessed the behavioral effects of roughness on auditory-tactile integration, which reflects the monitoring of this multisensory region of space. Healthy human participants had to detect as fast as possible a tactile stimulation delivered on their hand while an irrelevant sound was approaching them from the rear hemifield. The sound was either a simple harmonic sound or a rough sound, processed through binaural rendering so that the virtual sound source was looming towards participants. The rough sound speeded tactile reaction times at a farther distance from the body than the non-rough sound. This indicates that PPS, as estimated here via auditory-tactile integration, is sensitive to auditory roughness. Auditory roughness modifies the behavioral relevance of simple auditory events in relation to the body. Even without emotional or social contextual information, auditory roughness constitutes an innate threat cue that elicits defensive responses.

## Introduction

Auditory roughness was first described by Hermann von Helmholtz as the percept experienced when two sounds close in frequency are heard simultaneously^1^. Roughness can be produced by means of amplitude-modulated sounds, with the modulation frequency in the range of 20–200 Hz, for a given (non-zero) modulation depth. As already noted by Helmholtz, roughness is strongly linked to musical dissonance, and was generally studied within the field of music perception^2^. Roughness has also been found to be correlated to unpleasantness^3^, and is generally included in industrial measures of sound quality^4^.

The amplitude modulations underpinning perceived auditory roughness^4^ were found in artificial as well as in natural auditory alarms such as human screams^5^. They were also found in baby cries, especially those associated with high levels of distress^6^. Moreover, studies on human babies and on other species have shown that they can not only produce roughness but also perceive it, which suggests roughness as a basic auditory attribute, with a potential ecological validity^7,8^. It is indeed not plausible to interpret the existence of roughness perception in human babies, and even more in other species, as a result of a cultural construct^7,8^.

Auditory roughness, as featured in human screams, has been linked to an increased perception of fear that could be mediated by increased activation in the amygdala—a cerebral area involved in threat and danger processing^5^. Even simple non-semantic rough sounds (click trains, with the click train frequency varying between 50 and 250 Hz) influenced activity in several brain areas involved in aversion processing^9^. Auditory roughness thus seems to activate survival circuits in the perceiver’s brain. However, if it is the case, it should not only evoke negative emotions but it should induce innate behavioral responses, similar to those generated by threats to the integrity and survival of oneself or the offspring.

A rapid and accurate evaluation of danger and of the spatial location of the threat is crucial for the implementation of an appropriate behavior. In humans as in other animals, the proximity of a potential threat triggers defensive behaviors aiming at self-preservation^10–13^, and the activity of the amygdala and other brain areas of threat-related networks is modulated according to the egocentric distance of the danger source^14–16^. Furthermore, events in the proximal space are encoded in the brain distinctly from more distant events^17–20^. This space near and around the body has been called peripersonal space (PPS). It is selectively encoded in the brain by a neural network linked to multisensory integration processes^17,18,21–25^ and subserves a function in body protection and in the implementation of defensive behaviors^26^. PPS defines a graded field around the body, in which the behavioral relevance of external stimuli grows with their proximity^17,27^.

This far-to-near gradient of behavioral relevance has been repeatedly shown to be sensitive to threat in the environment. Several studies have contrasted the emotional valence of stimuli to examine the plasticity of PPS. They have reported that aggressive individuals^13,28,29^, alarming (screaming) individuals^30^, feared animals^31–34^, aversive and threatening objects^35^ or even unpleasant meaningless sounds (brown noises)^30^ approaching the body extend the graded field of PPS. This gives a greater margin of safety to devise appropriate actions.

We hypothesized that auditory roughness provides a threat cue sufficient to initiate rapid behavioral responses that can be measured through PPS assessment. The emotional valence of the stimulus should not be at stake; only the behavioral outcome should reveal the innateness of the auditory attribute. By acting directly on low-level mechanisms related to defensive responses, auditory roughness would then prove itself as a very important biological trait of communication.

In this study, we compared the behavioral effect on PPS of an approaching rough sound and an approaching non-rough sound. Several subjective ratings of the sounds were also collected: emotional valence and arousal, and perceived roughness.

We used the auditory-tactile interaction task developed by Canzoneri et al.^36^, based on the change in behavioral effects of multisensory integration as a function of stimuli proximity^37–40^. Participants have to perform a speeded tactile detection task while an irrelevant sound source is looming towards them. The tactile stimulation is delivered at different delays from the looming sound onset so that the sound source is perceived at different distances from participants’ body when the tactile stimulus is processed: at a far distance for short delays and at a closer distance for longer delays. The analysis of participants’ tactile reaction times (RTs) as a function of the delay from sound onset gives information about auditory-tactile integration processes around the body, and thus about PPS. Shorter RTs when the spatial distance between the body and the sound is diminishing show that the sound accelerates tactile processing. It indicates that the sound enters PPS and interacts with tactile processing. This method presents several advantages: first it provides an implicit and objective measure of sounds’ behavioral effect as it consists in measuring RTs to detect a stimulus in a given sensory modality while the stimulus of interest (the sound) is irrelevant to the task and presented in another sensory modality; second, it relies on behavioral consequences of multisensory integration that have been extensively studied^41–43^; and third, it uses dynamic approaching stimuli that constitute an intrinsic warning cue^44–46^.

We asked participants to perform the speeded tactile detection task in the presence of two meaningless sounds: a non-rough sound (harmonic sound) and a rough sound (the same harmonic sound amplitude-modulated at 70 Hz) looming towards them from the rear hemifield. We found that the looming rough sound started to speed up tactile RTs at a greater egocentric distance than the looming non-rough sound. This suggests that the rough sound was behaviorally relevant at a farther distance than the non-rough sound, extending the safety zone around the body to promote faster reaction if necessary.

## Methods

### Participants

Forty healthy individuals (21 women; age: *M* ± *SD* = 24.4 ± 4.2, range 18–37) with normal reported audition and touch participated in the study. All of them were right-handed. None of them had a history of psychiatric disorders, neurological disorders or was currently undergoing medical treatment.

All participants provided a written informed consent prior to the experiment, which was approved by the Institutional Review Board of INSERM (IRB00003888). The experiment was performed in accordance with the committee’s guidelines. Participants received a financial compensation of 20€ for their participation.

### Stimuli

The auditory stimuli were two simple synthetic sounds (called “non-rough sound” and “rough sound”) of 3000 ms duration (44,100 Hz sampling frequency). The non-rough sound was a harmonic sound with a fundamental frequency of 500 Hz, and 7 harmonics (up to 4 kHz). The relative amplitude of each harmonic was arbitrarily chosen (1 for the F0, 0.5 for F1, and around 0.25 for the other harmonics). The non-rough sound was amplitude-modulated (depth m = 1, modulation frequency f_m_ = 70 Hz) to generate the rough sound^4,47^. RTs are known to vary according to the loudness of the auditory stimuli^48^. We thus equalized the relative level of the two sounds to avoid any loudness difference between them. Moore et al.’s loudness model^49^ was used: a 0.8 dB difference between the two sounds was found (the rough sound being louder). To simulate the looming source, the sounds were processed in the Max/MSP (6.1.8) environment using the Spat library^50^. The direct sound, the first reflections up to the order 3, and a late reverberation were simulated. The spatialization of the direct sound and of the first reflections was rendered using non-individual head related transfer functions (HRTF) taken from the LISTEN HRTF database (http://recherche.ircam.fr/equipes/salles/listen/). To enhance the looming effect of the direct sound, binaural nearfield correction filters were also used^51^. The whole sound processing was applied to both the non-rough and the rough sound in order to render sound sources approaching from the rear hemifield from the left hemispace (135°), with a spatial location varying from 520 to 20 cm from the center of the participant’s head. The sound velocity was 166.67 cm s^−1^. None of the participants reported mislocalization or lack of externalization, and they all confirmed that they could hear the sounds approaching from the rear left hemispace.

The tactile stimulus was a vibratory stimulus delivered by means of a small loudspeaker on the palmar surface of the left index finger of participants. A sinusoidal signal was displayed during 20 ms at 250 Hz. We chose these parameters to insure that the vibration of the loudspeaker was perceivable, but that the sound was inaudible^33,52–54^.

### Apparatus

Participants sat on a chair in a soundproofed room. Their head was positioned on a headrest in order to minimize head movements during the experiment. They were blindfolded and their hands were positioned palms-down on a table. Participants were instructed to keep their hands aligned with their mid-sagittal plane and in contact with their trunk in order to study PPS around the trunk (and not PPS around the hand)^55^. Auditory stimuli were presented through Beyer Dynamic DT770 headphones. Tactile stimuli were presented by means of a 28 mm miniature loudspeaker (Veco, 8 Ω). Participants responded to tactile stimulation by pressing a key on a wired numeric keypad (Essentiel b). A PC running Presentation software (16.3 version—https://www.neurobs.com/) was used to control the presentation of the stimuli and to record the responses, with a precision of 1 ms for the RT.

### Design and procedure

To assess participants’ general level of anxiety, they were asked to complete the trait portion of the State Trait Anxiety Inventory (STAI^56^) before the experimental session (on a separate day). No participant presenting extremely high level of anxiety (score > 65 on the trait portion of the STAI) was included in the study. On the day of the experimental session, participants were first invited to complete the state portion of the STAI in order to evaluate their emotional state before the start of the protocol. Then, participants had to subjectively evaluate the emotional valence and arousal of the sounds used in the main experiment (the rough and the non-rough sounds). Then, they performed the main auditory-tactile interaction experiment allowing the behavioral measure of PPS. At the end of the experiment, participants had, again, to subjectively evaluate the emotional valence and arousal of the sounds. They also had to subjectively evaluate the roughness of the two sounds. Finally, they completed again the state portion of the STAI to assess any potential change in participants’ emotional state during the experimental session.

#### Subjective emotional evaluation task

To evaluate whether participants perceived the two sounds as emotionally charged and to assess any habituation phenomenon, participants performed a short emotional subjective evaluation task before and after the main experiment (emotional evaluation PRE and POST). This task design and method is the same as in previous work conducted with threatening sounds^33^. The two (non-spatialized) auditory stimuli were presented through headphones (at 76.5 dBA for the non-rough sound, and at 77.3 dBA for the rough sound; see “Stimuli” section for the loudness equalization). Each stimulus was presented only once. The order of stimuli presentation was counter balanced between subjects. Participants had their eyes closed during the display of the sounds. After the offset of the sound, they had to indicate, using a mouse, the perceived valence and arousal of the sound on digital vertical 10 cm visual analogic scales (VAS) displayed on a 13.3″ computer screen. The labels defining the end points of the valence VAS were “negative” and “positive”. The labels defining the end points of the arousal VAS were “no arousal” and “strong arousal”. Their responses were then translated into a score between 0 and 100.

#### Measure of PPS via the auditory-tactile interaction experiment

Participants completed an auditory-tactile interaction experiment that behaviorally assesses PPS. As in our earlier studies measuring PPS^33,52–54^, we used a modified version of Canzoneri et al.’s auditory-tactile interaction task^36^. During this task, participants have to press a button in response to tactile stimuli delivered on their hand while irrelevant looming sounds are played. RTs to tactile stimuli are measured to examine the behavioral consequence of the multisensory integration between the auditory and the tactile stimuli: shorter tactile RTs in presence of a sound is a signature of auditory-tactile integration. When the sound interacts with the tactile stimulus, it means that the sound entered participant’s PPS. The RTs typically vary as a function of the egocentric distance of the sound source: the smaller the sound source distance (i.e. longer delays), the shorter the tactile RTs^33,36,52–54^. In order to examine how a looming rough sound modifies PPS, tactile RTs have to be measured as the rough sound source is at different egocentric distances and then compared to the RTs measured with a non-rough looming sound.

**Figure 1:**
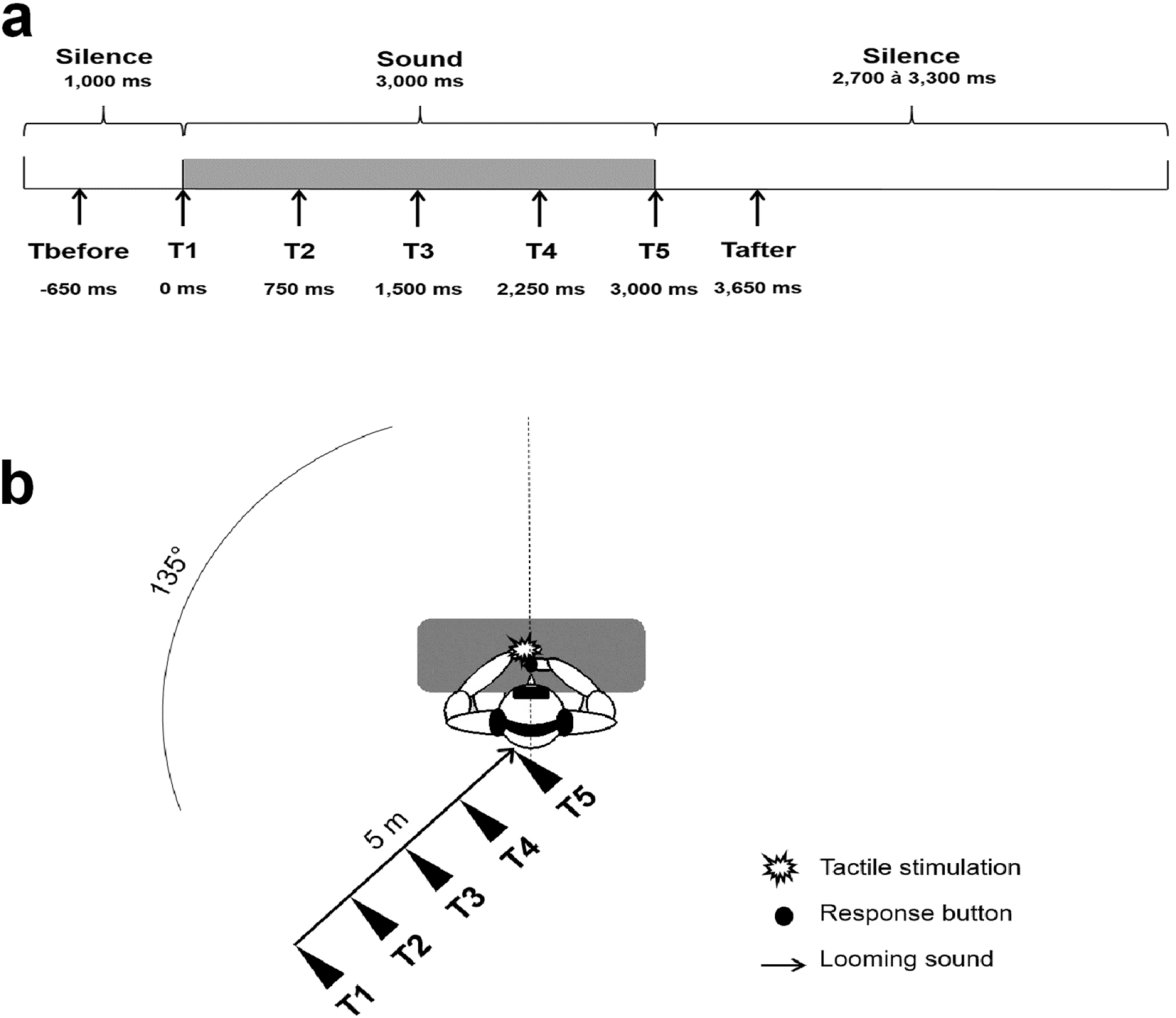
Auditory-tactile interaction experiment used for the estimation of PPS. (**a**) Description of a trial. A trial begins with a period of silence followed by a sound looming for 3000 ms and ends with a second period of silence. Along the trial, a tactile stimulation can occur at one among seven delays from sound onset (Tbefore, T1, T2, T3, T4, T5, Tafter). Delays are indicated as the time (in ms) between the sound onset and the tactile stimulation. (**b**) Experimental setup. Participants received a tactile stimulation on their left hand while task-irrelevant sounds (rough or non-rough) approached them from the left rear space. The tactile stimulus was delivered at different delays from sound onset. Hence, the sound source was perceived at different locations with respect to the body when tactile stimulation occurred: far from the body at small temporal delays and close to the body at long temporal delays (see filled black triangle).

An auditory stimulus was presented for 3000 ms in each trial (see Fig. 1). The sound source approached towards participants from the rear hemifield, from the left hemispace (135°) with a spatial location varying from 520 to 20 cm from the center of the participant’s head. Each auditory stimulus was preceded by 1000 ms of silence and followed by a silence with a duration varying between 2700 and 3300 ms^33,52–54^.

In 87.5% of the trials, a tactile stimulus was presented along with the auditory stimuli. The remaining 12.5% trials were catch trials with auditory stimulation only. Participants were instructed to ignore the auditory stimuli and to press a button with the index of their dominant (here: right) hand as quickly as possible each time a tactile stimulus was detected^33,52–54^.

Vibratory tactile stimuli were delivered at different delays from sound onset. With this procedure, the tactile stimuli were delivered when the sound source was perceived at varying distances from participants’ bodies. We examined at which delay the sound started to interact with tactile processing and speeded up tactile RTs^33,52–54^.

Temporal delays for the tactile stimulus were set as follows: T1 was a tactile stimulation delivered simultaneously with the sound onset; T2 at 750 ms from sound onset; T3 at 1500 ms from sound onset; T4 at 2250 ms from sound onset and T5 at 3000 ms from sound onset. Thus, tactile stimulation occurred when the sound source was perceived at different locations with respect to the body: far from the body at short temporal delays and close to the body at long temporal delays (see Fig. 1). Moreover, tactile stimulations were also delivered during the silent periods, preceding or following the sound, at – 650 ms (T_before_) before the sound onset and at + 650 ms (T_after_) after the sound offset (i.e. 3650 ms after the sound onset)^33,52–54^. The RTs measured in these unimodal conditions give an insight into the changes in RTs which are not linked to multisensory integration effects^53,57^. For most of the trials, a tactile stimulus occurred at varying delays from the sound onset; thus, the probability of tactile stimulus occurrence increased along with the sound duration. This expectancy effect can speed up RTs but their influence can be accounted for^53,57^ (see Supplementary Information).

A small training block (about 2 min) was first ran to familiarize participants with the task. Then, participants were requested to describe the sound they perceived. All the participants correctly perceived the direction of sound movement as well as the changes in sound source distance. The total experimental test consisted of 20 repetitions of each of the 14 conditions, presented in a random order. The factors were: DELAY (seven levels: T_before_, T1, T2, T3, T4, T5 and T_after_) and SOUND TYPE (two levels: non-rough/rough). There were a total of 280 trials with a tactile target, randomly intermingled with 40 catch trials. Trials were equally divided into 10 blocks of 32 trials, lasting about 4 min each.

#### Subjective roughness evaluation task

To verify that participants actually perceived the modulated sound as rougher than the unmodulated sound, participants also performed a subjective roughness evaluation task after the auditory-tactile interaction experiment. We explained to participants that auditory roughness is similar to tactile roughness, i.e. a quality of the sound opposed to smoothness; a rough sound is a sound that grips. The procedure for the subjective roughness evaluation task was similar to that of the emotional evaluation task except that the end points of the digital vertical 10 cm VAS were “a little rough” and “extremely rough”.

### Statistical analysis

Except when specified, all the statistical tests conducted and reported hereinbelow were two-tailed with an alpha level of 0.05 and were conducted on the data of the 40 participants. For each analysis, we assessed the distribution of the data of interest and conducted non-parametric statistical tests when the distribution deviated from normality. We assessed the deviation from normality with Kolmogorov–Smirnov tests, when necessary. For the analysis of RTs with ANOVAs, the amounts of data, on which the deviation from normality was studied, were very high (10,891 RT data). Consequently, the power of the statistical tests was so high that the tests would detect a significant deviation from the normal distribution even though the deviation was, in fact, small. Together with the facts that “moderate departures from normality are not usually fatal” to ANOVAs^58^ and that visual inspection of our data distribution was coherent with a normal distribution, this allowed us to consider that our data did not deviate enough from normality to forbid the use of parametric tests.

## Results

### Participants’ characteristics

The participants’ scores on the trait portion of the STAI ranged from 25 to 61 (*M* ± *SD* = 38.8 ± 8.7) and thus covered a large part of the range of possible scores (20 to 80). The distribution of the trait STAI scores did not significantly deviate from a normal distribution (Kolmogorov–Smirnov test, *p* > 0.20). The distribution of the state STAI scores did not significantly deviate from a normal distribution either (Kolmogorov–Smirnov test, *p* > 0.20 for both the scores before and after the experiment). There was no significant difference between participants’ scores on the state portion of the STAI before and after the experiment (respectively *M* ± *SD* = 28.2 ± 6.6 and *M* ± *SD* = 27.9 ± 6.9; Student test: t_(39)_ = 0.30, *p* = 0.768).This shows that there was no significant change in participants’ emotional state during the experimental session.

### Subjective emotional evaluation task

We conducted an ANOVA separately on the valence and arousal scores reported by the participants with the within-subject factors SOUND TYPE (two levels: rough/non-rough) and TIME (two levels: emotional evaluation PRE/emotional evaluation POST).

**Figure 2:**
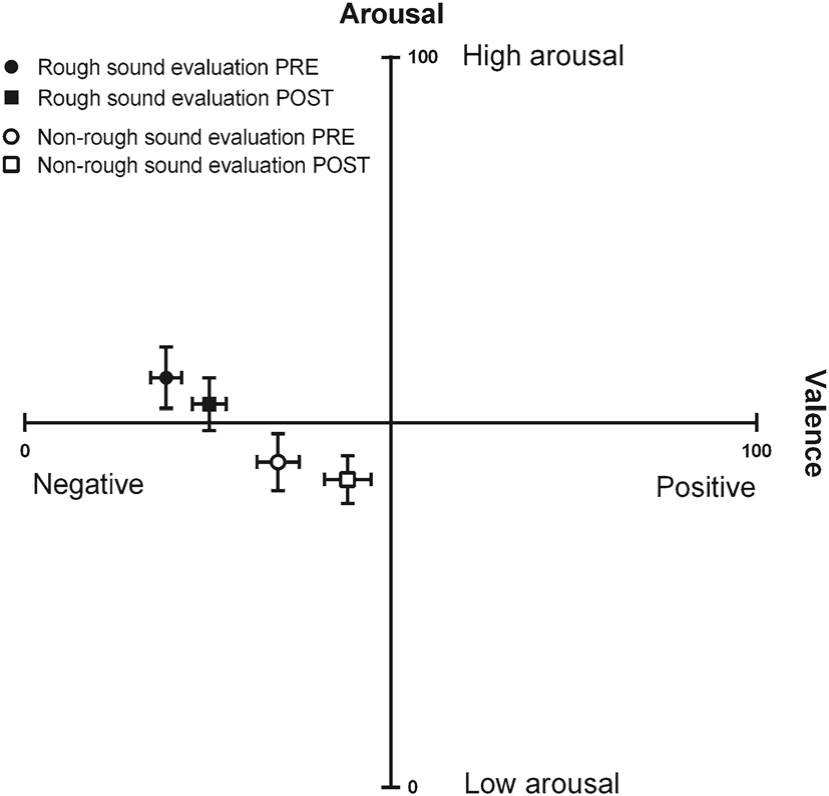
Subjective emotional evaluation task results. This figure depicts the perceived arousal and valence scores (mean ± SEM) reported by participants (n = 40) in the emotional evaluation PRE (before the auditory-tactile interaction experiment) in response to the rough (filled black circle) and non-rough (open circle) sound and in the emotional evaluation POST (after the auditory-tactile interaction experiment) in response to the rough (filled black square) and non-rough (open square) sound. Both sounds were perceived as negatively-valenced. The valence of the rough sound was slightly more negative than the valence of the non-rough sound in both emotional evaluations. The rough sound was also perceived as slightly more arousing than the non-rough sound in both emotional evaluations.

The main effect of SOUND TYPE on valence scores was significant (*F*(1, 39) = 39.32, *p* < 0.001, η_*p*_^2^ = 0.502). As shown in Fig. 2, in both emotional evaluations PRE and POST, participants perceived the rough sound as more negatively valenced than the non-rough sound. The main effect of SOUND TYPE on arousal scores was also significant (*F*(1, 39) = 20.36, *p* < 0.001, η_*p*_^2^ = 0.343). In both emotional evaluations, participants perceived the rough sound as more arousing than the non-rough sound. These results indicate that the rough sound was perceived as more arousing and more negative than the non-rough sound. However, this does not mean that the non-rough sound was perceived as positively-valenced. In both emotional evaluations, both the rough and non-rough sounds were perceived as negative, with scores significantly lower than 50 (one sample Student test, one tailed: p < 0.035 and Cohen’s d > 0.29 in all four cases).

The main effect of TIME on valence scores was significant (*F*(1, 39) = 11.71, *p* = 0.001, η_*p*_^2^ = 0.231). Both sounds were perceived as less negatively valenced after the experiment than before (POST vs PRE). However, the main effect of TIME on arousal scores was not significant (*F*(1, 39) = 1.06, *p* = 0.337) suggesting that both sounds were perceived as arousing before and after the experiment.

The effect of the two-way interaction SOUND TYPE*TIME was not significant on either the valence or the arousal scores (p > 0.207 in both cases) suggesting that the changes in the emotional evaluation of the auditory stimuli across time were not different according to whether the sound was rough or not. The emotional habituation phenomena occurring during the experiment were not different between the two sounds.

### PPS measure via auditory-tactile interaction experiment

The processing and analysis of the data collected during the auditory-tactile experiment were conducted with the same procedure as in our previous studies^33,52–54^. As attested by the very low rate of false alarms and omissions (0.5% and 0.5% respectively), participants were extremely accurate. Consequently, the performances were only analyzed in terms of RTs. The lack of precision in RT measures due to interruptions from operating systems or device drivers were removed from the analyses (2.1% of the trials). RTs shorter than 100 ms or exceeding 1000 ms were also removed from the analyses (0.2% of the trials) because we considered these RTs too short or too long to correspond to participants properly performing the speeded tactile detection task. The remaining data were averaged for each participant, for each SOUND TYPE condition and each DELAY condition.

We conducted an ANOVA on the mean RTs of participants with the within-subject factors SOUND TYPE (two levels: rough/non-rough) and DELAY (7 levels: T_before_, T1, T2, T3, T4, T5, T_after_). The mean RTs in each condition are reported in the Supplementary information (Table S1), along with the standard deviation and the 95% confidence interval of the mean RTs. There was no significant main effect of SOUND TYPE (*F*_*(1, 39)*_ = 0.13, *p* = 0.726). The main effect of DELAY was significant (*F*_*(6, 234)*_ = 81.41, *p* < 0.001, η_*p*_^2^ = 0.676). The two-way interaction SOUND TYPE*DELAY was also significant (*F*_*(6, 234)*_ = 2.29, *p* = 0.036, η_*p*_^2^ = 0.056). This suggests that tactile RTs were differently influenced by the perceived distance of the sound source, depending on the roughness of the sound.

**Figure 3:**
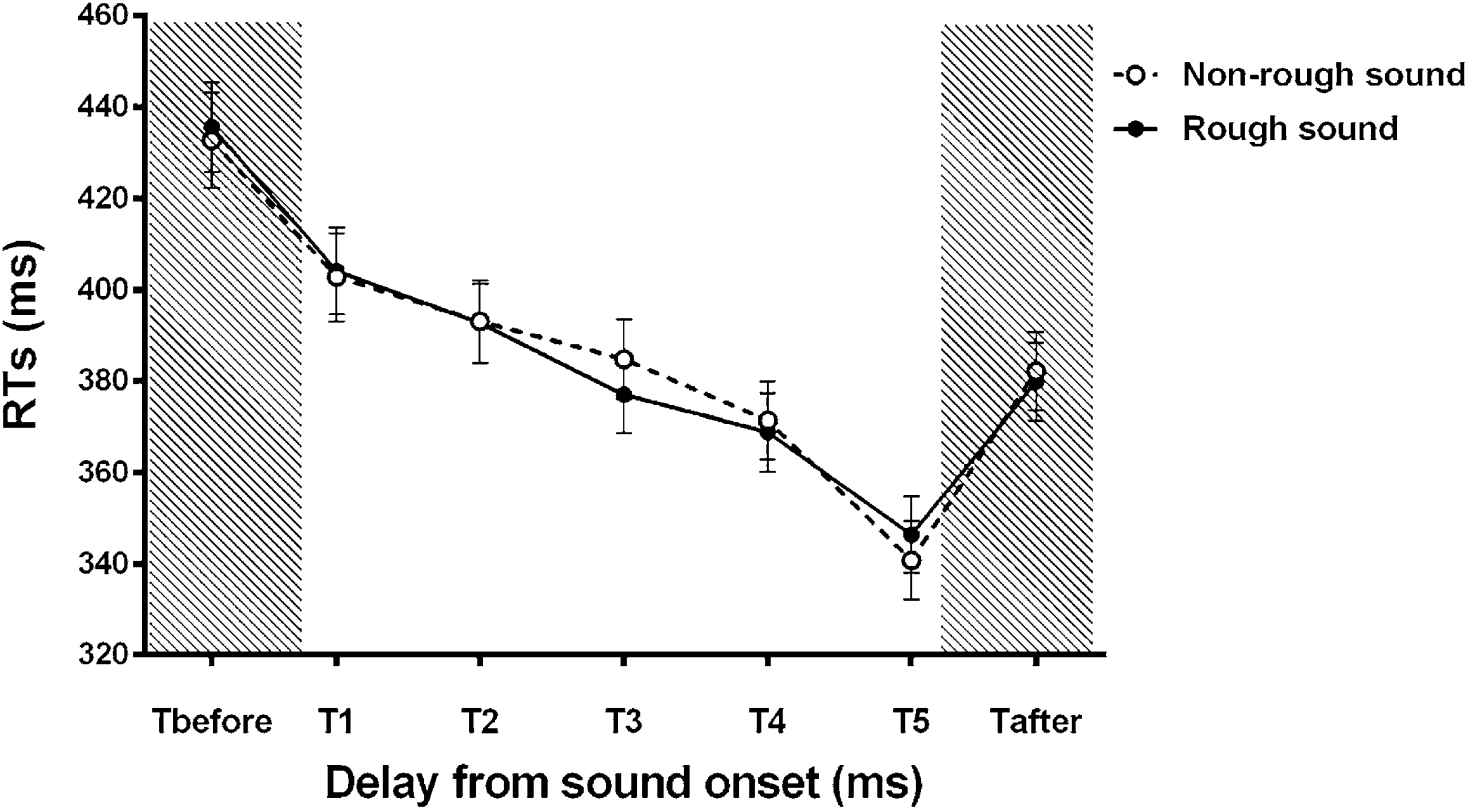
Auditory-tactile interaction experiment results—Analysis of mean reaction times (RTs). This figure depicts participants’ (n = 40) mean tactile reaction times (RTs ± SEM) in the rough (filled black circle) and non-rough (open circle) sound conditions, as a function of the delay between the tactile stimulation and the sound onset. The shaded regions indicate the silent periods during which the tactile stimulation occurred alone either before sound onset or after sound offset (unimodal conditions). Auditory-tactile integration is evidenced by shorter RTs; here, the first significant decrease in RTs occurred at T3 for the rough sound, and T5 for the non-rough sound, with T3 corresponding to a farther perceived sound distance. It means that efficient auditory-tactile integration occurred at a farther sound distance from the body for rough sound, thus suggesting a larger PPS for rough sounds.

As can be seen on Fig. 3, when the sound was not rough, the first significant decrease of participants’ RTs occurred when the tactile stimulus was delivered at T5 (long delay, i.e. short perceived sound distance). RTs at T5 were significantly shorter than RTs at T4 (post hoc Scheffé test: *p* < 0.001; Cohen’s d = 1.06) whereas there were no significant differences between RTs at T1 and T2 (post hoc Scheffé test: *p* = 0.569), RTs at T2 and T3 (post hoc Scheffé test: *p* = 0.802) or RTs at T3 and T4 (post hoc Scheffé test: *p* = 0.063). Moreover, participants’ RTs were significantly shorter when the tactile stimulus occurred at T5 as compared to when the tactile stimulus was delivered at T1, T2, T3 (post hoc Scheffé test: *p* < 0.001; Cohen’s d > 1.32 in all cases).

Contrastingly, when the sound was rough, the first significant decrease of participants’ RTs occurred when the tactile stimulus was delivered at T3. RTs at T3 were significantly shorter than RTs at T2 (post hoc Scheffé test: *p* = 0.006; Cohen’s d = 0.84) whereas there were no significant differences between RTs at T1 and T2 (post hoc Scheffé test: *p* = 0.268). Moreover, participants’ RTs were significantly shorter when the tactile stimulus occurred at T3, T4 and T5 as compared to when the tactile stimulus was delivered at T1, T2 (post hoc Scheffé test: *p* < 0.006; Cohen’s d > 1.00 in all cases). RTs also further decreased between T4 and T5 (post hoc Scheffé test: *p* < 0.001; Cohen’s d = 0.87).

Overall, these results suggest that the rough sound began to affect tactile RTs at a farther distance than the non-rough sound.

RTs in the unimodal condition T_before_ were significantly longer than RTs in the bimodal conditions T1, T2, T3, T4 and T5, in both sound type conditions (post hoc Scheffé test: *p* < 0.001; Cohen’s d > 1.10 in all cases), evidencing audio-tactile integration. Furthermore, in both sound type conditions, RTs in the unimodal condition T_after_ were significantly shorter than RTs at T_before_ (post hoc Scheffé test: *p* < 0.001; Cohen’s d > 1.39 in both cases). These results suggest that other effects than multisensory integration play a role in the observed changes in tactile RTs along delay conditions. The difference in tactile RTs between T_before_ and T_after_ can be explained by the increasing probability of receiving a tactile stimulation along trials^53,57^. However, given that RTs at T_after_ were significantly longer than RTs at T5 (post hoc Newman-Keuls test: *p* < 0.001; Cohen’s d > 1.28 in both sound type conditions), we can exclude the possibility that participants’ faster RTs in the bimodal condition at late delays are solely explained by tactile expectation. Participants’ shorter RTs could rather be explained by both tactile expectations and multisensory integration. We also analyzed our data on the basis of the findings of Hobeika et al.^53^, to take these expectancy effects into account. This analysis, which is reported in the Supplementary Information (Supplementary Data Analyses, Table S2 and Fig. S2), revealed similar results as the one described above. This strongly suggests that expectancy effects alone cannot explain our pattern of results.

**Figure 4:**
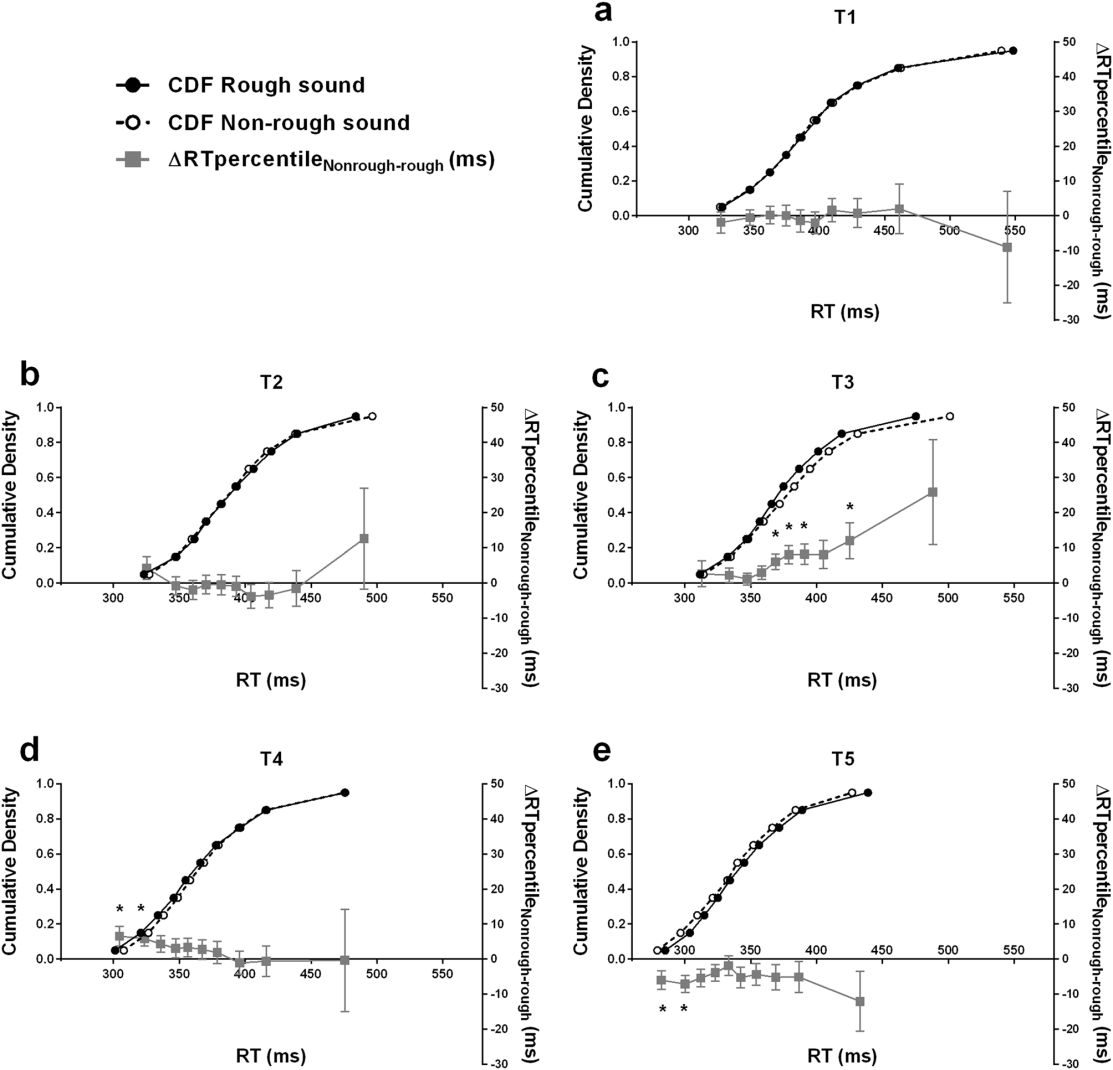
Auditory-tactile interaction experiment results—Analysis of reaction times (RTs) distribution. This figure depicts the participants’ (n = 40) average cumulative density function (CDF) of RTs at the delays T1, T2, T3, T4, T5 (respectively **a**–**e**) in the non-rough (open circle) and rough (filled black circle) sound conditions. For easier visual assessment, the mean difference between the RT percentile values in the non-rough and rough sound conditions (ΔRTpercentile_Nonrough-Rough_ ± SEM) at each bin is also plotted (filled grey square). The significant differences are indicated with an asterisk. At delays T1 and T2, i.e. at far sound distances from the body, there was no significant difference in RTs distribution between the two sound conditions. At T3 the median and median-to-late RTs were faster in the rough sound condition. At T4 the early RTs were faster in the rough sound condition. At T5 the early RTs were slower in the rough sound condition compared to the non-rough sound condition. These results confirm even more clearly the conclusion: auditory-tactile integration was more efficient at a farther sound distance with the rough sound than with the non-rough sound. This strongly suggests that the PPS extends for the rough sounds as compared to non-rough sounds.

To complete our analysis on mean RTs, we conducted a distributional RT analysis. We used the algorithm and MATLAB code described by Ulrich et al.^59^ to compute the average cumulative density function (CDF) of RTs for each delay condition and each sound type. First, the empirical CDF for each condition of delay and sound type was estimated for each participant. Then, percentile values were computed from these empirical CDFs. We used ten bins, from the 0.05 percentile to the 0.95 percentile with an increment of 0.1. Finally, the RT percentile values for each of the ten bins were aggregated across participants to obtain the average CDF reflecting participants’ RTs distribution. These CDFs are presented, along with the corresponding delta plots, on Fig. 4, for each sound type and for delays T1, T2, T3, T4 and T5 (CDFs and delta plots for delays Tbefore and Tafter are provided in the Supplementary Information on Fig. S3). The delta plots enable an easier visual comparison of two CDFs by plotting the difference between RT percentile values in two experimental conditions^60^.

In order to compare RT distributions in the rough and non-rough sound condition, we performed bin by bin paired t-tests on the RT percentile values in both conditions, separately for each delay. We used the Benjamini–Hochberg correction to control for multiple testing^61^. We did not observe any significant difference between CDFs in the rough and in the non-rough sound conditions for delays Tbefore, T1, T2 and Tafter (*p* > 0.061 in all cases). Contrastingly, we observed a difference between CDFs for delays T3, T4 and T5. At T3, RT values for percentiles 0.45, 0.55, 0.65 and 0.85 were significantly different between the rough and the non-rough sound condition (*p* < 0.038, Cohen’s d > 0.37 in all cases). RT percentiles values were shorter in the rough condition (see Fig. 4c). The RT values for the other percentiles did not differ between sound conditions (*p* > 0.101 in all cases). At T4, RT values for percentiles 0.05 and 0.15 were significantly different between the rough and the non-rough sound condition (*p* < 0.027, Cohen’s d > 0.38 in both cases). RT percentiles values were shorter in the rough sound condition (see Fig. 4d). The RT values for the other percentiles did not differ between sound conditions (*p* > 0.109 in all cases). Finally, at T5, RT values for percentiles 0.05 and 0.15 were also significantly different between the rough and the non-rough sound condition (*p* < 0.042, Cohen’s d > 0.35 in both cases). Contrastingly, here, the RT percentiles values were larger in the rough sound condition (see Fig. 4e). The RT values for the other percentiles did not differ between sound conditions (*p* > 0.059 in all cases).

In summary, at T3, the median and median-to-late RTs were faster in the rough sound condition; at T4, the early RTs were faster in the rough sound condition; and at T5, the early RTs were slower in the rough sound condition, compared to the non-rough sound condition.

### Subjective roughness evaluation task

Participants’ responses on the Roughness VAS were not normally distributed (Kolmogorov-Smirnoff tests, *p* < 0.01 for both the rough and non-rough sound VAS scores). Hence, we compared the roughness scores between the two sounds using a non-parametric test (Wilcoxon test for matched samples). The roughness scores of the rough sound (median = 82.40, 1st quartile = 66.95, 3rd quartile = 89.65, min = 19.10, max = 100.00) were significantly higher than the roughness scores of the non-rough (median = 17.95, 1st quartile = 5.70, 3rd quartile = 35.80, min = 0.00, max = 78.10) sound condition (T = 11.00, p < 0.001, effect size: r = 0.85, n = 40). In accordance with the literature, this result confirms that the ‘rough sound’ was actually perceived as rougher than the ‘non-rough sound’.

## Discussion

We investigated whether auditory roughness can provoke defense reactions in humans. We evaluated the behavioral responses to rough and non-rough sounds in healthy humans using an implicit and objective measure. This measure consists in the examination of the influence of a sound stimulus on the reaction time to a tactile stimulus delivered on the hand, as a function of the sound source distance. It allows a psychophysical assessment of the far-to-near gradient of the sound behavioral relevance. Although both the rough and the non-rough sound were perceived as negatively-valenced, participants’ response to the tactile stimuli were speeded up by the rough sound at a farther egocentric distance than with the non-rough sound. The rough sound started to boost participants’ RTs at distances corresponding to delay conditions T3 and T4. In comparison, the non-rough sound improved detection performance only when positioned at a closer distance from participants’ body (delay T5). To sum up, the auditory attribute of roughness in a looming sound extended participants’ PPS, shrinking far space closer to the level of near space and improving behavioral efficiency.

The present results are consistent with previous work reporting a relationship between roughness and the aversive quality of sounds^5,6,9^. They bring further the behavioral examination of the auditory attribute of roughness, measured with meaningless sounds. Here, the roughness effect seems particularly robust despite the subtle indirect behavioral measure we used, in a sample of participants with normal and homogeneous levels of state anxiety, and with a contrasting non-rough sound that was already perceived as unpleasant. This indicates that auditory roughness constitutes an innately aversive attribute.

Similar modifications of the behavioral effects of auditory-tactile integration along egocentric distances have been reported in the literature with stimuli such as dog growling sounds and women screams^30,33^ that were contrasted with neutral or positively valenced sounds (sheep bleating and baby laughing sounds). These threatening sounds also speeded up tactile RTs at a farther distance than non threatening ones. This change in the spatial map of stimuli relevance is thought to act as an increase of the margin of safety for the self in the presence of a threat and to allow additional time for the preparation of defensive behaviors^26^. Yet, the fact that the rough sound was not contrasted in terms of valence with the non-rough sound implies that the behavioral effect we found is not linked to the subjective emotional value of auditory roughness. The simple auditory attribute of roughness seems to be sufficient to cause threat-induced like behavioral modifications—for that matter, it is also very possible that it contributes to the PPS modifications found in response to growling sounds and screams, since these stimuli sound rough too.

The behavioral advantage given by the auditory roughness gives a greater margin of safety to implement appropriate defensive action^26^. However, one result, found in the CDF analysis, suggested that at the closest distance to the body, the rough sound seemed less efficient than the non-rough sound to accelerate tactile detection. If confirmed by future studies, this relationship between the roughness effect and the distance to the body could be a new way to study the functional defense behavior system in humans^62^.

The auditory attribute of roughness has been shown to correlate with stronger activation in the amygdala and with enhanced neural synchronization in subcortical and cortical areas involved in emotion processing^5,9^. Although the understanding of the cerebral correlates of PPS multisensory encoding is growing^17,21,26,63–66^, the neural bases of the dynamic and instantaneous modifications of PPS by a threatening event remain unknown to date. Nonetheless, the processing of auditory roughness might proceed through the amygdala in a rather bottom-up fashion to modulate, in the end, the receptive fields of the fronto-parietal multisensory neurons encoding PPS in order to make them respond to farther stimuli. Future research examining this question may contribute to the body of work investigating the relationship between affective and multisensory processes^67–69^ by unravelling whether, how, when and where affective and multisensory processes interact depending on the proximity of threatening events.

Here, the efficient multisensory integration in PPS was measured with auditory and tactile stimuli. The neural substrates for audition and somatosensation are anatomically linked^70^, have common ontogenetic origin^71^, and share representations of frequency^72^. The strong link between touch and sounds (we produce sound when we explore an object with the hands) might further promote the efficient detection of a tactile stimulus in presence of a looming sound. Several experiments have already demonstrated that a looming visual stimulus facilitates the detection of a tactile stimulus when approaching the body^32,57^. Nonetheless, it remains to be tested if the spatial proximity of a rough sound can also enhance detection in different sensory modalities.

The “roughness effect” found in this study seems particularly interesting from an applied point of view, for the design of warning sounds, used in a large variety of environments (military or civilian planes, operating rooms in hospitals, cars, …). The user should detect and react as fast and accurately as possible to these warning sounds^73^. The use of natural sounds, or even more useful, of synthetic sounds based on the acoustical attributes derived from the behavioral advantage of natural warning sounds has also been suggested in the past^74^. Here, we evidenced a clear behavioral advantage conferred by auditory roughness. The use of roughness is a good starting point to design warning sounds that exploit natural enhancement of defense reactions.

Auditory roughness is an efficient attribute to convey signals of danger without the need of additional context, independently of the anxiety level of the perceiver. Even when the auditory attribute of roughness is deprived of any information about the environment, it generates adaptive behavior. In the present experiment, roughness acts as an innate threatening auditory stimulus modulating defensive responses measured through auditory-tactile integration in peripersonal space. Studies on babies and other species^7,8^ have shown that they are sensitive to auditory roughness, suggesting that its innately aversive attribute could be universal. Auditory roughness constitutes an important attribute to study the biological basis of complex communicative behavior.

## Supplementary Information

Supplementary Information.
