# Supplementary material for: Auditory roughness elicits defense reactions
Source: Sci Rep. 2021 Jan 13;11:956. doi: 10.1038/s41598-020-79767-0 (PMC7806762; doi:10.1038/s41598-020-79767-0)
Supplement: Supplementary file 1 — Supplementary Information. [file 41598_2020_79767_MOESM1_ESM.docx]

**Supplementary Information**

**Auditory roughness elicits defense reactions**

Marine Taffou^1*^, Clara Suied^1^, Isabelle Viaud-Delmon^2^

^1^ Institut de Recherche Biomédicale des Armées, 91220 Brétigny-sur-Orge, France

^2^ CNRS, Ircam, Sorbonne Université, Ministère de la Culture, Sciences et Technologies de la Musique et du son, STMS, F-75004 Paris, France

^*^Corresponding author: marine.taffou@gmail.com

**Supplementary Table S1**

| Table S1. Participants’ tactile reaction times in the auditory-tactile experiment (mean, standard deviation and confidence interval) | | | |
| --- | --- | --- | --- |
| **Sound Type** | **Delay** | **Mean ± SD** | **95% CI** |
| Non-rough Sound |  |  |  |
|  | Tbefore | 432.70 ± 66.38 | [411.47 ; 453.93] |
|  | T1 | 402.73 ± 60.90 | [383.26 ; 422.21] |
|  | T2 | 393.06 ± 57.67 | [374.62 ; 411.51] |
|  | T3 | 384.72 ± 55.53 | [366.96 ; 402.48] |
|  | T4 | 371.34 ± 53.92 | [354.10 ; 388.59] |
|  | T5 | 340.77 ± 54.63 | [323.30 ; 358.24] |
|  | Tafter | 382.11 ± 54.12 | [364.80 ; 399.42] |
| Rough Sound |  |  |  |
|  | Tbefore | 435.64 ± 62.28 | [415.73 ; 455.56] |
|  | T1 | 404.02 ± 60.22 | [384.76 ; 423.28] |
|  | T2 | 392.68 ± 54.82 | [375.15 ; 410.21] |
|  | T3 | 376.96 ± 52.06 | [360.31 ; 393.61] |
|  | T4 | 368.77 ± 54.54 | [351.33 ; 386.22] |
|  | T5 | 346.33 ± 53.19 | [329.32 ; 363.34] |
|  | Tafter | 379.88 ± 53.95 | [362.62 ; 397.13] |

**Supplementary Data Analysis**

To have a better insight on the effects of the sounds on tactile RTs, and to disentangle genuine multisensory integration from various other possible biases, we further analyzed and transformed our data in order to take the expectancy effects into account. As recently shown by Hobeika et al. (2019), in auditory-tactile interaction tasks such as the one used in the present study, expectation of receiving a tactile stimulation increases with time during trials and accelerates tactile RTs. This effect of expectation grows linearly with time when the possible delays of tactile stimulation are distributed on a linear scale^53^. Thus, in order to take expectancy effects into account, we estimated the decrease of RTs due solely to expectancy effects as the difference between RTs for the two extreme delays conditions, Tbefore (tactile stimulus occurred before the sound onset) and Tafter (tactile stimulus occurred after the sound offset). RT data were normalized with respect to this expectancy effect estimation. For each participant and for each sound type condition (non-rough/rough), we fitted a linear function with the RTs measured at T_before_ and T_after_. The linear function was described by the following equation: $y\left( x \right)=a*x+y_{0}$ where $x$ represents the independent variable (i.e. the delay of the tactile stimulation from sound onset in ms), $y$ the dependent variable (i.e. tactile RT in ms), $a$ is the slope and $y_{0}$ is the intercept at $x=0$. The distribution of the slopes and the intercepts did not significantly deviate from a normal distribution (Kolmogorov-Smirnov test, *p*> 0.20 for both parameters and for both sounds). These two parameters were not significantly different according to the sound type condition (slope: *M_nonrough_ ± SD =* -0.012 *±* 0.008, *M_rough_ ± SD =* -0.013 *±* 0.009, Student test: *t(39)* = 1.06, *p*=0.293 ; intercept: *M_nonrugh_ ± SD =* 436.82 *±* 68.09, *M_rough_ ± SD =* 440.18 *±* 64.03, Student test: *t(39)* = -0.88, *p*= 0.385). We then again fitted a linear function with the RTs measured at T_before_ and T_after_ for each participant, but this time with the data from both sound type conditions pooled together. From these individual linear functions, we calculated for each participant and for each delay the theoretical decrease of tactile RTs due to expectancy effects. This value was then subtracted from the measured tactile RTs, in each corresponding delay and participant and for each sound type condition. These corrected tactile RTs (cRTs) are displayed in Figure S2b. Analyses of the cRTs should provide a more direct examination of the contribution of PPS-related multisensory integration effects on tactile detection.

Similar analyses as for the non-corrected RTs (figure S2a) were performed on the cRTs (figure S2b). Overall, as described below, results were similar for these cRTs as for the non-corrected RTs, thus showing that, although present, expectancy effects are not responsible for the observed efficient auditory-tactile integration.

We conducted an ANOVA on the mean cRTs of participants, with the within-subject factors SOUND TYPE (two levels: rough/non-rough) and DELAY (7 levels: T_before_, T1, T2, T3, T4, T5, T_after_). There was no significant main effect of SOUND TYPE (*F_(1, 39)_* = 0.13, *p* = 0.726). The main effect of DELAY was significant (*F_(6, 234)_* = 47.802, *p* < 0.001, η*_p_*^2^ = 0.551). The two-way interaction SOUND TYPE*DELAY was also significant (*F_(6, 234)_* = 2.29, *p* = 0.036, η*_p_*^2^ = 0.056) suggesting that cRTs were differently influenced by the temporal delays, depending on the roughness of the sound. As shown on Figure 3b, for the non-rough sound, the first significant decrease of participants’ cRTs occurred when the tactile stimulus was delivered at T5. cRTs at T5 were significantly shorter than cRTs at T4 (post hoc Fisher’s LSD test: *p* < 0.001 ; Cohen’s d = 1.75) whereas there were no significant differences between cRTs at T1 and T2 (post hoc Fisher’s LSD test: *p* = 0.878), cRTs at T2 and T3 (post hoc Fisher’s LSD test: *p* = 0.752) or cRTs at T3 and T4 (post hoc Fisher’s LSD test: *p* = 0.149). Moreover, participants’ cRTs were significantly faster when the tactile stimulus occurred at T5 as compared to when the tactile stimulus was delivered at T1, T2 and T3 (post hoc Fisher’s LSD test: *p* < 0.001; Cohen’s d > 0.83 in all cases).

Contrastingly, when the sound was rough, the first significant decrease of participants’ cRTs occurred when the tactile stimulus was delivered at T3. cRTs at T3 were significantly shorter than cRTs at T2 (post hoc Fisher’s LSD test: *p* = 0.024; Cohen’s d = 0.36) whereas there were no significant differences between cRTs at T1 and T2 (post hoc Fisher’s LSD test: *p* = 0.462). Moreover, participants’ cRTs were significantly or tended to be shorter when the tactile stimulus occurred at T3, T4 and T5 as compared to when the tactile stimulus was delivered at T1 and T2 (post hoc Fisher’s LSD test: *p* < 0.024; Cohen’s d > 0.30 in all cases except *p*= 0.059; Cohen’s d = 0.24 when comparing cRTs at T2 to cRTs at T4). RTs also further decreased between T4 and T5 (post hoc Fisher’s LSD test: *p* < 0.001; Cohen’s d = 1.53). Finally, cRTs at T3 were significantly shorter in the rough sound condition than in the non-rough sound condition (post hoc Fisher’s LSD test: *p* = .007; Cohen’s d = 0.45). These results suggest that the rough sound began to affect tactile RTs at a farther distance than the non-rough sound.

Furthermore, there is, as expected in this ANOVA on cRTs, no significant difference between the cRTs at T_before_ and T_after_ neither between or inside the sound type conditions (post hoc Fisher’s LSD test: *p* > 0.302 in all cases).

**Supplementary Table S2**

| Table S2. Participants’ corrected tactile reaction times in the auditory-tactile experiment (mean, standard deviation and confidence interval) | | | |
| --- | --- | --- | --- |
| **Sound Type** | **Delay** | **Mean ± SD** | **95% CI** |
| Non-rough Sound |  |  |  |
|  | Tbefore | 432.70 ± 66.38 | [411.47 ; 453.93] |
|  | T1 | 410.76 ± 63.19 | [390.55 ; 430.97] |
|  | T2 | 410.32 ± 62.56 | [390.31 ; 430.33] |
|  | T3 | 411.22 ± 63.35 | [390.96 ; 431.48] |
|  | T4 | 407.09 ± 63.50 | [386.79 ; 427.40] |
|  | T5 | 385.77 ± 60.03 | [366.57 ; 404.97] |
|  | Tafter | 435.12 ± 65.10 | [414.30 ; 455.94] |
| Rough Sound |  |  |  |
|  | Tbefore | 435.64 ± 62.28 | [415.73 ; 455.56] |
|  | T1 | 412.04 ± 62.49 | [392.06 ; 432.03] |
|  | T2 | 409.94 ± 59.34 | [390.96 ; 428.92] |
|  | T3 | 403.46 ± 58.36 | [384.80 ; 422.12] |
|  | T4 | 404.52 ± 62.40 | [384.57 ; 424.48] |
|  | T5 | 391.32 ± 58.64 | [372.57 ; 410.08] |
|  | Tafter | 432.89 ± 62.80 | [412.80 ; 452.97] |

**Supplementary Figure S2**


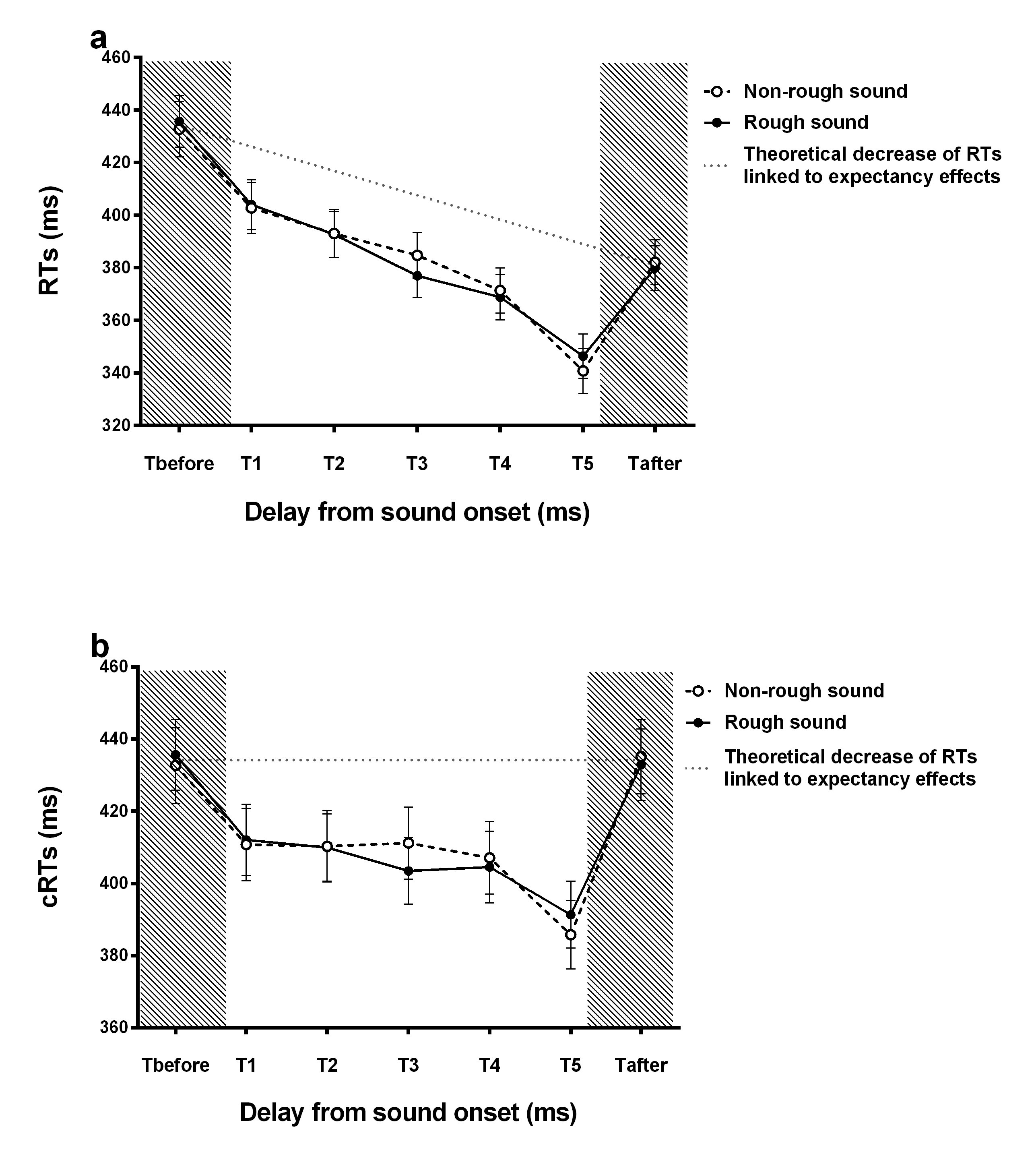


Figure S2. Auditory-tactile interaction experiment results - Analysis of mean reaction times (RTs) and corrected reaction times (cRTs). (a) This figure depicts participants’ (n=40) mean tactile reaction times (RTs ± SEM) in the rough ( ) or non-rough ( ) sound conditions as a function of the delay of tactile stimulation delivery from sound onset (the longer the delay, the closer the sound source). The shaded regions indicate the silent periods during which the tactile stimulation occurred alone either before sound onset or after sound offset. The dotted line represents the theoretical decrease of RTs linked to expectancy effects. (b) This figure depicts participants’ (n=40) mean tactile reaction times normalized with respect to the estimation of expectancy effects for each participant and each condition (cRTs ± SEM). Results are similar as with the non-corrected RTs, showing that expectancy effects alone cannot explain the pattern of results: the sound distance for which tactile reaction times were faster was farther from the body with the rough sound than with the non-rough sound. The rough sound extended PPS.

**Supplementary Figure S3**


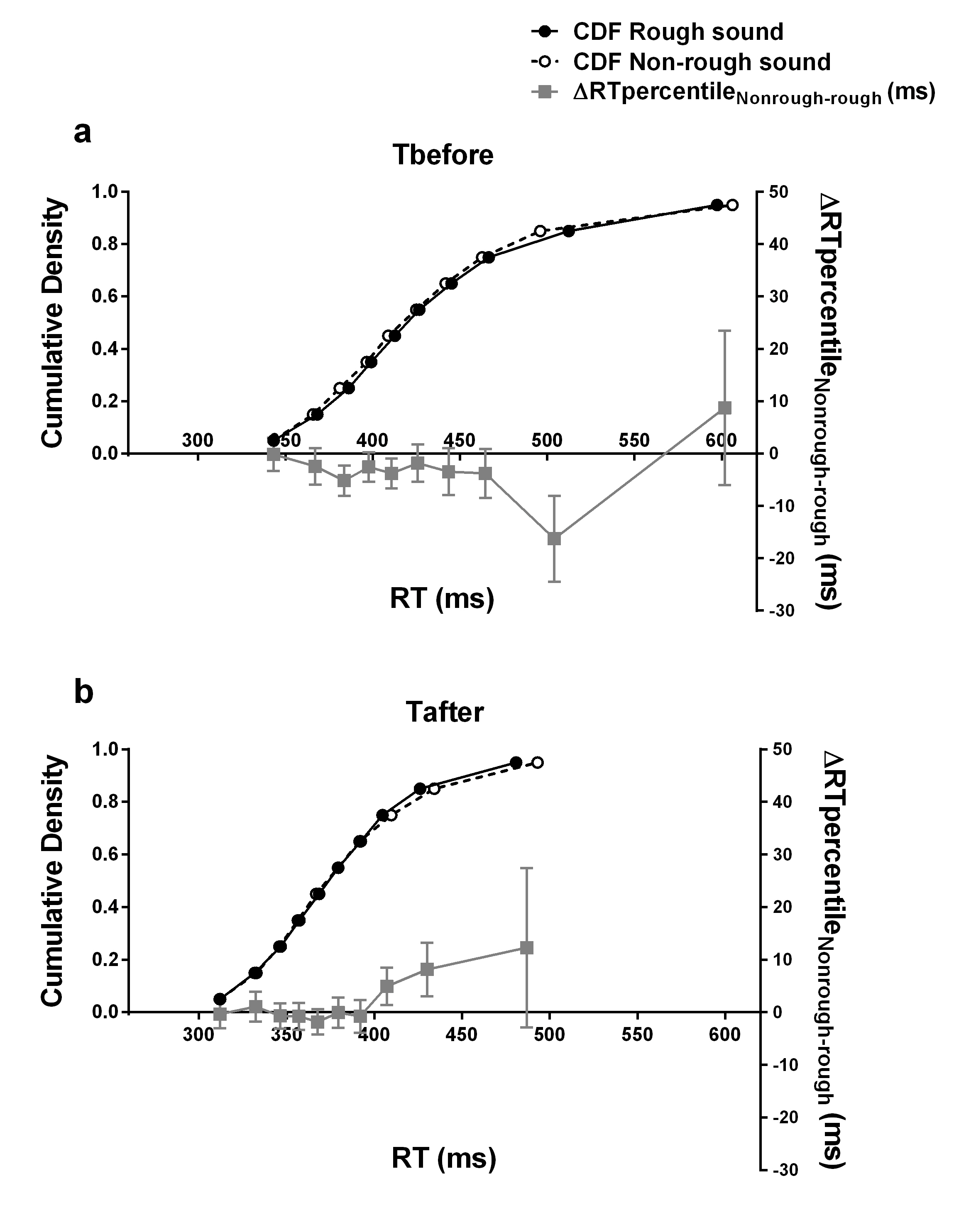


Figure S3. Auditory-tactile interaction experiment results - Analysis of reaction times (RTs) distribution in the unimodal delay conditions. This figure depicts the average cumulative density function (CDF) of RTs at the delays Tbefore (a) and Tafter (b) in the non-rough ( ) and rough ( ) sound conditions. The mean difference between the RT percentile values in the non-rough and rough sound conditions (ΔRTpercentile_Nonrough-Rough_ ± SEM) at each bin is also plotted ( ). For the delays Tbefore and Tafter, there was no significant difference in RTs distribution between the two sound conditions.
